# The Importance of Psychological Assessment in the Management of Bladder Pain Syndrome/Interstitial Cystitis and Vulvodynia: A Case Report

**DOI:** 10.7759/cureus.63617

**Published:** 2024-07-01

**Authors:** Nobuo Okui, Machiko A Okui

**Affiliations:** 1 Dentistry, Kanagawa Dental University, Kanagawa, JPN; 2 Urology, Yokosuka Urogynecology and Urology Clinic, Kanagawa, JPN; 3 Urogynecology, Yokosuka Urogynecology and Urology Clinic, Kanagawa, JPN

**Keywords:** sexual aversion disorder scale, toronto alexithymia scale-20, generalized anxiety disorder-7, patient health questionnaire-9, dyspareunia, vulvodynia, bladder pain syndrome/interstitial cystitis

## Abstract

This case report emphasizes the crucial role of psychological assessment in the management of patients with bladder pain syndrome/interstitial cystitis (BPS/IC) and vulvodynia. A 48-year-old woman with a five-year history of refractory BPS/IC and vulvodynia presented with frequent urination, pelvic pain, and severe dyspareunia, which led to sexual aversion and divorce from her partner. Previous treatments, including lifestyle modifications, analgesics, anticholinergics, hydrodistension, intravesical dimethyl sulfoxide, and psychiatric interventions, had been ineffective. Psychological assessments using the Patient Health Questionnaire-9, Generalized Anxiety Disorder-7, and Toronto Alexithymia Scale-20 revealed severe symptoms of depression, anxiety, and alexithymia. Due to the patient’s sexual aversion and the absence of a partner, a complete Female Sexual Function Index (FSFI) could not be administered. Instead, a partial FSFI and artificial intelligence-translated reference value of the Female Sexual Distress Scale-Revised were used to assess aspects relevant to the patient’s condition. The patient underwent three monthly sessions of Fotona laser therapy, erbium, and neodymium laser at one-month intervals. Treatment outcomes were evaluated using the Numeric Rating Scale-11, Vulvodynia Total, Interstitial Cystitis Symptom Index, and psychological assessment tools. At the six-month follow-up, all physical and psychological symptoms showed significant improvement and complete remission was achieved at 12 months. Despite the overall positive treatment outcomes, the patient’s sexual aversion persisted, and accurate measurement was not possible, highlighting the complexity of addressing sexual function in patients with BPS/IC and vulvodynia. This case report underscores the need for a holistic approach to managing these conditions, addressing both the physical and psychological aspects of the disease.

## Introduction

Bladder pain syndrome/interstitial cystitis (BPS/IC) is a chronic condition characterized by pelvic pain and urinary symptoms that significantly impact patients’ quality of life [1,2]. Despite the established diagnostic criteria and treatment guidelines for BPS/IC [1,3,4], the heterogeneity of symptoms and the presence of comorbidities, such as vulvodynia, may not be adequately captured by validated questionnaires [5,6].

Recent studies have demonstrated the potential of laser therapy, particularly Fotona Laser which is a combination of erbium:YAG (VEL) and neodymium:YAG (Nd:YAG) lasers, as a promising treatment option for vulvodynia and BPS/IC [5-10].

However, no studies have yet demonstrated that the improvement in vulvodynia symptoms following laser treatment is accompanied by a significant improvement in psychological symptoms. Determining whether addressing the physical aspects of vulvodynia may have a positive impact on mental well-being is a crucial question that remains to be answered.

In this case report, we present a 48-year-old woman with a five-year history of refractory BPS/IC and vulvodynia. The patient underwent a comprehensive psychological assessment, including the use of a partial Female Sexual Function Index (FSFI) [11] due to the absence of a partner, which revealed severe psychological distress related to her condition. The patient received treatment with a combination of vaginal erbium laser and neodymium:YAG laser therapy. This case highlights the importance of a holistic approach in the management of BPS/IC and vulvodynia, with a particular emphasis on the assessment and treatment of psychological factors.

## Case presentation

A 48-year-old healthy female patient, who was para 1 with one spontaneous vaginal delivery 17 years prior, with a body mass index of 25.2 kg/m², presented to our clinic with a five-year history of frequent urination and pelvic pain. The patient complained of regular increasing suprapubic pain during urination and throughout the day. She experienced severe pain during intercourse, which led to divorce from her partner. She expressed a strong aversion to sexual intercourse. Lifestyle modifications, various analgesics, oral hydroxyzine treatment, and anticholinergic (oxybutynin and trospium) treatments were ineffective. A cystoscopy performed at another medical center detected no lesions in the bladder, and random biopsy results showed no malignant lesions, only findings of interstitial inflammation. Furthermore, hydrodistension performed simultaneously with cystoscopy did not provide any relief. A trial of DMSO bladder instillation was ineffective.

Findings at our clinic

Physical examination revealed normal findings in the urogenital system. No cystocele, rectocele, or prolapse was detected, and stress test results were negative. The vulvodynia swab test revealed pain at the 2, 10, and 12 o'clock positions of the hymenal remnants, with Visual Analog Scale (VAS) scores of 10, 9, and 7 (0, no pain; 10, maximum pain).

Blood count and biochemistry tests showed no abnormalities. Urinalysis results were normal, and the urine culture showed no bacterial growth. Urine cytology and cervical smear test results were negative. Uroflowmetry showed a maximum flow rate of 17 mL/second, a voided volume of 156 mL, and no detectable post-void residual urine. Data from a three-day voiding diary indicated that the patient urinated every 60-70 minutes. The average daily fluid intake over the three days was 1,800, 2,200, and 2,300 mL, respectively. Bladder neurological testing (urodynamic testing, pressure-flow study) could not be performed because the insertion of the testing catheter (4 Fr) into the urethra provoked severe pain. Abdominal ultrasonography did not reveal uterine fibroids. The ovaries had no cysts or other lesions.

Fotona laser treatment

Combined VEL and Nd:YAG laser treatments, known as Fotona laser therapy, were conducted in an outpatient setting. The patient visited our clinic for the first laser treatment (T0), and VEL + Nd:YAG was administered once a month for three months. After the final laser treatment, follow-up was conducted at six months (T06) and 12 months (T12). The evaluations were performed at T0, T06, and T12.

**Figure 1:**
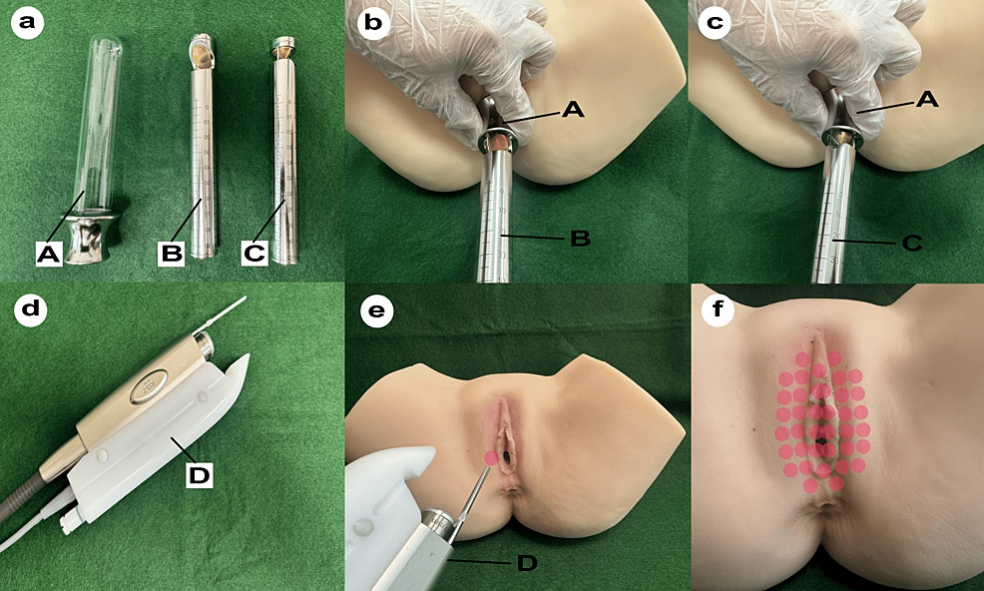
Fotona laser treatment with combined VEL and Nd:YAG laser treatments. a: The laser tools. A: a glass speculum; B: handpiece PS03-GA; C: handpiece R11-GC1. b: Laser irradiation targeted to the anterior wall. c: Laser irradiation targeted to the 360 wall. d: The laser tools. D: R33 non-contact handpiece. e: The irradiation process using an R33. f: The irradiation sites.

Figure 1 illustrates the accessories and application techniques used in the Fotona laser treatment. Figure 1a shows the tools used for VEL, including a glass speculum (A) inserted into the vagina, handpiece PS03-GA (B) for irradiating only the anterior vaginal wall, and handpiece R11-GC1 (C) to irradiate the entire vaginal circumference. Figure 1b shows the insertion of the PS03-GA (B) handpiece into a model after inserting the glass speculum (A), washing the patient’s vagina with a disinfectant, and drying it with a cotton swab before treatment. The RenovaLase® VEL protocol (laser irradiation at a wavelength of 2,940 nm) is shown. Figure 1c shows the insertion of the R11-GC1 (C) handpiece into the model after insertion of the glass speculum (A). To prevent tissue ablation due to deep thermal effects in the vagina, the laser spot diameter was set to 7 mm, the frequency to 1.6 Hz, and the pulse fluence to 1.75 J/cm^2^. Figure 1d shows the R33 non-contact handpiece (D) used for the Nd:YAG laser (Fotona SP Dynamis, PIANO mode, spot size of 9 mm, PIANO pulse mode (five seconds), fluence of 90 J/cm2). Figure 1e shows the irradiation process using an R33 (D) handpiece. Figure 1f indicates the irradiation sites. Patients were advised to avoid sexual intercourse for one week after each session.

Assessment tools for BPS/IC with comorbid vulvodynia

For BPS/IC with comorbid vulvodynia, validated assessment tools were used, including the Numerical Rating Scale-11 (NRS-11) [5-9], vulvodynia swab test [5-9], Interstitial Cystitis Symptom Index (ICSI) [5-9], Interstitial Cystitis Problem Index (ICPI) [5-9], Pelvic Pain and Urgency/Frequency (PUF) [5-9], Overactive Bladder Questionnaire Short Form (OABq SF) [5-9], Overactive Bladder Symptom Score (OABSS) [5-9], and Pelvic Floor Distress Inventory-20 (PFDI-20) [5-9]. The vulvodynia swab test was performed at the 2, 4, 8, and 10 o'clock positions on the vaginal vestibule, and the level of pain at each site was evaluated using the VAS (0, no pain; 10, worst pain). The total scores from the four sites were used in the analysis. Table 1 shows the changes in scores. The scores that were high at T0 showed significant improvement after the three laser treatments over a year.

**Table 1:** Assessment tools for BPS/IC with comorbid vulvodynia. BPS/IC: bladder pain syndrome/interstitial cystitis; NRS-11: Numerical Rating Scale-11; ICSI: Interstitial Cystitis Symptom Index; ICPI: Interstitial Cystitis Problem Index; PUF: Pelvic Pain and Urgency/Frequency; OABq SF: Overactive Bladder Questionnaire Short Form; OABSS: Overactive Bladder Symptom Score; PFDI-20: Pelvic Floor Distress Inventory-20; T0: before laser treatment; T6: six months after the third laser treatment; T12: 12 months after the third laser treatment

Assessment tool	T0	T6	T12
NRS-11	7	4	0
Vulvodynia swab test	30	8	0
ICSI	6	4	0
ICPI	6	3	0
PUF	12	9	0
OABq SF	15	4	0
OABSS	6	3	0
PFDI-20	102	82	0

Psychological evaluation

Standardized questionnaires and psychological tests were administered by a specialist (psychologist-trained nurse) in a relaxed environment. As shown in Table 2, a total score of 14 on the Patient Health Questionnaire-9 (PHQ-9) [12] indicated moderate depression, with the patient reporting feelings of depression, loss of interest, sleep disturbances, fatigue, poor appetite, concentration difficulties, and thoughts of self-harm.

**Table 2:** Patient Health Questionnaire-9. T0: before laser treatment; T6: six months after the third laser treatment; T12: 12 months after the third laser treatment

Question item	T0	T6	T12
a. Little interest or pleasure in doing things	2	1	1
b. Feeling down, depressed, or hopeless	1	1	0
c. Trouble falling or staying asleep, or sleeping too much	1	1	0
d. Feeling tired or having little energy	2	1	0
e. Poor appetite or overeating	2	2	0
f. Feeling bad about yourself - or that you are a failure or have let yourself or your family down	1	1	1
g. Trouble concentrating on things, such as reading the newspaper or watching television	1	1	0
h. Moving or speaking so slowly that other people could have noticed? Or the opposite - being so fidgety or restless that you have been moving around a lot more than usual	2	1	0
i. Thoughts that you would be better off dead or of hurting yourself in some way	2	1	0
Total	14	10	2

As shown in Table 3, the total score of 26 on the Generalized Anxiety Disorder-7 (GAD-7) scale [13] suggested severe anxiety, with the patient experiencing nervousness, uncontrollable worry, restlessness, and irritability.

**Table 3:** Generalized Anxiety Disorder-7. T0: before laser treatment; T6: six months after the third laser treatment; T12: 12 months after the third laser treatment

Question item	T0	T6	T12
a. Feeling nervous, anxious, or on edge	4	2	0
b. Not being able to stop or control worrying	4	2	0
c. Worrying too much about different things	4	2	0
d. Trouble relaxing	4	2	0
e. Being so restless that it is hard to sit still	4	2	0
f. Becoming easily annoyed or irritable	4	2	1
g. Feeling afraid, as if something awful might happen	2	2	0
Total	26	14	1

As shown in Table 4, the scores on the Toronto Alexithymia Scale (TAS) [14] indicated high levels of alexithymia, a condition characterized by difficulty in identifying and describing emotions. The patient scored 24 on the Difficulty Describing Feelings (TAS-DDF) subscale, 12 on the Difficulty Identifying Feelings (TAS-DIF) subscale, and 28 on the Externally Oriented Thinking (TAS-EOT) subscale, indicating significant difficulties in emotional processing and a tendency to focus on external rather than inner experiences.

**Table 4:** Toronto Alexithymia Scale-20. T0: before laser treatment; T6: six months after the third laser treatment; T12: 12 months after the third laser treatment

Subscale	T0	T6	T12
Difficulty Describing Feelings (TAS-DDF)	24	20	9
Difficulty Identifying Feelings (TAS-DIF)	12	18	7
Externally Oriented Thinking (TAS-EOT)	28	21	12
Total	64	59	28

These psychological findings suggested that the patient’s BPS/IC score and vulvodynia symptoms were likely influenced by underlying depression, anxiety, and alexithymia. Depression and anxiety contribute to central sensitization and increase pain perception. Alexithymia may hinder a patient’s ability to recognize and communicate emotional distress, leading to increased pain and psychological distress. The patient received treatment from a psychiatrist, including the administration of antidepressants, such as selective serotonin reuptake inhibitors or serotonin-norepinephrine reuptake inhibitors, but they were ineffective. In cognitive-behavioral therapy, the patient was instructed to keep a diary, but this was also ineffective.

Owing to the patient’s sexual aversion stemming from the divorce episode and her lack of a current sexual partner, the complete FSFI was deemed to be psychologically burdensome and not fully applicable. Therefore, a modified version of the FSFI focusing on items that could be answered without a partner was administered to assess the patient’s sexual function and pain-related symptoms. The selected FSFI items and scores at T0, T6, and T12 are presented in Table 5.

**Table 5:** Selected Female Sexual Function Index. T0: before laser treatment; T6: six months after the third laser treatment; T12: 12 months after the third laser treatment

Item	T0	T6	T12
Frequency of sexual desire or interest	1	1	1
Level of sexual desire or interest	1	1	1
Frequency of sexual arousal	0	0	0
Level of sexual arousal	0	0	0
Difficulty with lubrication during sexual activity	0	0	0
Frequency of pain during sexual activity	0	0	0
Level of pain during sexual activity	0	0	0

The Female Sexual Distress Scale-Revised (FSDS-R) [15] was adopted because the FSFI does not accurately assess patients’ aversion to sex. However, as there is no Japanese version of the FSDS-R, a translation provided by ChatGPT was used. Therefore, the values should not be considered as reference values. Table 6 shows the progress of FSDS-R scores at T0, T6, and T12.

**Table 6:** Female Sexual Distress Scale-Revised. T0: before laser treatment; T6: six months after the third laser treatment; T12: 12 months after the third laser treatment

Item	T0	T6	T12
1. Distressed about your sex life	4	3	1
2. Unhappy about your sexual relationship	4	4	2
3. Guilty about sexual difficulties	4	3	2
4. Frustrated by your sexual problems	4	3	2
5. Stressed about sex	4	3	2
6. Inferior because of sexual problems	2	2	2
7. Worried about sex	4	3	2
8. Sexually inadequate	4	3	2
9. Regrets about your sexuality	4	3	2
10. Embarrassed about sexual problems	4	3	2
11. Dissatisfied with your sex life	4	3	2
12. Angry about your sex life	4	3	2
13. Bothered by low sexual desire	4	3	2
Total	52	38	28

T6 and T12 outcomes

At T6, six months after the three laser treatments, the patient’s physical symptoms showed significant improvement. NRS-11, vulvodynia swab test, ICSI, ICPI, PUF, OABq SF, OABSS, and PFDI-20 scores decreased, indicating a reduction in pain, urinary urgency, frequency, and pelvic floor distress. The scores on the PHQ-9, GAD-7, and TAS-20 also showed improvement trends, but moderate-to-severe symptoms of depression, anxiety, and alexithymia were still observed. The selected FSFI items remained unchanged from the baseline, with low frequency and level of sexual desire or interest and no reported sexual arousal, lubrication, or sexual activity. The FSDS-R scores at T6 showed a slight improvement compared to baseline, with most scores decreasing from 4 to 3, indicating a reduction in distress related to sexual problems. However, the patient still reported feeling inferior because of sexual problems (score 2) and experienced distress, unhappiness, and other negative emotions related to her sexual life.

At T12, 12 months after the three laser treatments, the patient’s physical symptoms showed further improvement, with most scores reaching zero or near-zero levels. The NRS-11, vulvodynia swab test, ICSI, ICPI, PUF, OABq SF, OABSS, and PFDI-20 scores were all significantly lower than baseline, indicating a substantial reduction in pain and urinary symptoms. The scores on each psychological assessment scale improved remarkably. The PHQ-9 score was 2 points and the GAD-7 score was 1 point, indicating that depression and anxiety symptoms had almost disappeared. The total score on the TAS-20 decreased significantly to 28 points, indicating improvements in identifying and describing emotions and externally oriented thinking tendencies. However, the selected FSFI items remained the same as the baseline and T6 assessments, suggesting that the patient’s sexual function did not improve significantly despite improvements in her physical and psychological symptoms related to BPS/IC and vulvodynia. The FSDS-R scores at T12 showed further improvement, with most scores decreasing to 2, and the patient reported less distress regarding her sex life (score 1). Nevertheless, the patient still experienced negative emotions and distress related to her sexual problems and low sexual desire.

These findings suggest that Fotona laser treatment for BPS/IC with comorbid vulvodynia may have long-term effects on both physical and psychological symptoms, including pain, urinary symptoms, depression, anxiety, and alexithymia. The FSDS-R results indicated that the patient’s distress related to sexual problems improved over time but did not completely resolve. Issues related to sexual function persisted after treatment, possibly because of the patient’s lack of sexual partners and ongoing sexual aversion. Further psychological support and sex therapy should be considered if the patient desires to address her sexual concerns in the future.

## Discussion

A previous study [10] revealed that some patients with BPS/IC had unhealthy external genitalia, and the BPS/IC group had worse vulvar pain swab test and total Vaginal Health Index score compared to the control group (11.59 ± 2.87 vs. 22.05 ± 3.05, p < 0.05). However, the characteristics of patients with BPS/IC and comorbid vulvodynia are not well understood. An unsupervised machine learning study [9] using standardized BPS/IC questionnaires identified three phenotypic clusters, one of which had a high prevalence of comorbid vulvodynia. This cluster was characterized by high vulvodynia pain scores, whereas the OABSS and OABq SF scores were moderate. The present case exhibited features consistent with the previously reported vulvodynia-predominant subtype [9], characterized by high vulvodynia pain scores and moderate OABSS and OABq SF scores.

The psychological characteristics of patients with BPS/IC and vulvodynia are not well known. However, Ricucci et al. [16] reported on the psychological state of vulvodynia. They investigated 372 vulvodynia patients and revealed a high prevalence of psychological problems, such as depression, anxiety, and alexithymia. Specifically, the PHQ-9 [12] assessment showed that the depression score of the vulvodynia group (Group 1) was significantly higher at 11.36 ± 5.21 compared to the other groups. The GAD-7 [13] assessment revealed that the anxiety score of Group 1 was significantly higher at 19.86 ± 7.36 compared to the cured group (Group 4). In contrast, the TAS-20 [14] showed no significant differences in alexithymia scores among the groups. The present case also showed high scores on the PHQ-9 (14 points), GAD-7 (26 points), and TAS-20 (64 points) before treatment, which is consistent with the psychological characteristics of patients with vulvodynia reported by Ricucci et al. These results suggest that it is important to focus not only on physical symptoms but also on psychological problems, particularly depression and anxiety, and provide appropriate evaluation and intervention for patients with BPS/IC and comorbid vulvodynia.

FSFI [11] is commonly used to evaluate sexual function. In the past, research on sexual aversion was conducted, and when sexual aversion was listed in the Diagnostic and Statistical Manual of Mental Disorders in 1987, sex therapist Helen Singer Kaplan reported the characteristics of 373 patients with sexual aversion [17]. According to the report, 19% of patients who avoided sexual intercourse met the criteria for panic disorder, and 25% of women who avoided sexual intercourse and had a phobia of sex also had comorbid panic disorder. In 1989, Katz et al. used a 30-item questionnaire on sexual aversion [18]; however, the concept was unified into sexual dysfunction, and the FSFI was used for patients with BPS/IC and vulvodynia.

In the present case, the patient had BPS/IC and comorbid vulvodynia, and during a period of extreme pain, her partner demanded sexual intercourse, which eventually led to a divorce. The patient also expressed an aversion to sexual intercourse; therefore, it was judged that the FSFI survey should be conducted with caution. However, the patient did not attempt to resolve her aversion to sex from the mental stress she had experienced in the past. This suggests the limitations of the commonly used FSFI. Although the FSDS-R was used to assess the patient’s sexual distress, it failed to accurately capture the specific nature of her sexual aversion.

Another available questionnaire is the Sexual Quality of Life Questionnaire-Female [19] which comprehensively evaluates the quality of sexual life and considers the impact of dyspareunia and sexual trauma. However, these questionnaires appear to be inadequate for accurately reflecting the sexual aversion experienced by patients with BPS/IC and vulvodynia.

For these patients, it is necessary to develop and introduce an evaluation method specific to sexual aversion, rather than relying solely on existing questionnaires. A flexible survey using artificial intelligence should be conducted to better understand and address the unique challenges faced by patients with sexual aversion in the context of BPS/IC and vulvodynia.

Regarding laser therapy, Okui et al. first reported the effectiveness of VEL in patients in 2019 [6]. VEL was found to be effective in improving bladder pain. In 2022, Butrick et al. reported the effects of TV-PBM in IC/BPS patients [8].

The energy carried by laser irradiation reacts with moisture in the mucosa to generate heat, thereby achieving a surface regeneration effect. VEL therapy for the vagina effectively changes the vaginal epithelium in patients with severe vaginal atrophy, leading to recovery of the vaginal epithelium and urethral mucosa, epithelial metabolic cycle, glycogen loading, papillomatosis, increased collagen formation, and increased cell density in the lamina propria. Long et al. [20] also showed that Er:YAG vaginal laser treatment had improved sexual function and vaginal tightening in women with stress urinary incontinence, as evidenced by the FSFI questionnaire and three-dimensional transperineal ultrasound. Although these reports did not mention comorbid vulvodynia, the high prevalence of vulvodynia in patients with BPS/IC suggested that a common pain mechanism involving the vagina and bladder might be involved.

Furthermore, a case report demonstrated that combination therapy with VEL and Nd:YAG laser improved symptoms in a patient with both vulvodynia and BPS/IC, and tissue regeneration was observed in biopsies of both the bladder and vagina [7]. In 2023, a case series demonstrating the effectiveness of VEL and Nd:YAG combination therapy was published [5].

In the present case, three sessions of VEL and Nd:YAG combination therapy resulted in a significant improvement in both BPS/IC and vulvodynia symptoms. Psychological assessments also showed substantial decreases in depression, anxiety, and alexithymia scores, demonstrating both physical and psychological effects. However, sexual function did not improve after treatment. This may be due to the patient’s sexual aversion and absence of a partner.

## Conclusions

Based on a case report of a 48-year-old woman with refractory BPS/IC and vulvodynia, combined VEL and Nd laser therapy resulted in significant improvements in both physical symptoms and psychological distress, including reductions in depression, anxiety, and alexithymia scores. However, despite these positive outcomes, the patient’s sexual function did not improve due to persistent sexual aversion influenced by past negative experiences. This case highlights the necessity of a holistic approach that addresses both physical and psychological aspects and suggests the development of specific evaluation methods for sexual aversion to enhance treatment outcomes for patients with BPS/IC and vulvodynia.
